# Maturation Differentially Regulate Protein Kinase C-Mediated BK Channel Activation in Ovine Middle Cerebral Artery

**DOI:** 10.64898/2025.12.03.692156

**Published:** 2025-12-07

**Authors:** Michell Goyal, Ravi Goyal

## Abstract

Evidence shows that fetal cerebral vessels are more prone to rupture than adult vessels during a sudden rise in systemic blood pressure (BP), leading to intraventricular hemorrhages and brain damage. Our research indicates that fetal cerebral arteries rely more on calcium-independent pathways for contraction than adult arteries, with Protein Kinase C (PKC) playing a key role in these pathways. In this study, we tested the hypothesis that PKC differentially regulates K+ channel activity in fetal and adult cerebral arteries, focusing on large-conductance calcium-activated K+ (BK) and ATP-sensitive (KATP) channels.

Using segments of the middle cerebral arteries (MCA) from near-term fetal and nonpregnant adult sheep, we measured vascular tension and intracellular calcium in response to a PKC agonist with and without selective K+ channel blockers. We found that PKC induced contraction without increasing intracellular calcium in both fetal and adult MCA. However, in fetal MCA, blocking BK channels increased PKC-induced calcium influx and contraction, while in adult MCA, BK channel inhibition did not affect calcium influx. Other K+ channel blockers (KATP, Kv, KIR) had no effect in either group.

These results suggest that in fetal MCA, PKC regulates vascular tone by activating BK channels to limit calcium influx, a mechanism not observed in adults. This highlights a developmental-specific role of PKC in modulating BK channel activity in the fetal cerebral vasculature.

## Full Text Availability

The license terms selected by the author(s) for this preprint version do not permit archiving in PMC. The full text is available from the preprint server.

